# Protocol for minicircle production for gene therapy without subsequent cleanup steps

**DOI:** 10.1016/j.xpro.2025.103982

**Published:** 2025-07-24

**Authors:** Jonathan Do, Gautam Verma, Yasmin Maurice, Muniba Abdumanobova, Zicheng Deng, Tanya V. Kalin, Vladimir V. Kalinichenko

**Affiliations:** 1 Phoenix Children’s Research Institute, University of Arizona College of Medicine-Phoenix, Phoenix, AZ, USA; 2 Center for Cancer and Blood Disorders, Phoenix Children’s Hospital, Phoenix, AZ 85016, USA; 3 Department of Child Health, Division of Hematology and Oncology, University of Arizona College of Medicine-Phoenix, Phoenix, AZ 85004, USA; 4 Division of Neonatology, Phoenix Children’s Hospital, Phoenix, AZ 85016, USA

**Keywords:** Biotechnology and bioengineering, Gene Expression, Microbiology

## Abstract

Current techniques for producing minicircles have low yields because of the cleanup steps required. Here, we present a protocol that can increase minicircle yield up to 10-fold by eliminating post-induction cleanup steps. We describe steps for bacterial culture, same-day arabinose induction, and DNA harvesting. This protocol is relevant for all minicircles produced from parental plasmids containing attB, attP, and 32 copies of Isce-I endonuclease sites in the minicircle-producing bacteria, ZYCY10PS3T2.

## Highlights

• Minicircle harvesting protocol optimized up to 10–20x the original protocol
• Enhanced minicircle purity by eliminating cleanup steps that reduce total yield
• Preparation is the same as a typical maxiprep harvesting kit

## Abstract (editor highlights)

Publisher’s note: Undertaking any experimental protocol requires adherence to local institutional guidelines for laboratory safety and ethics.

## Graphical abstract

**Figure:**
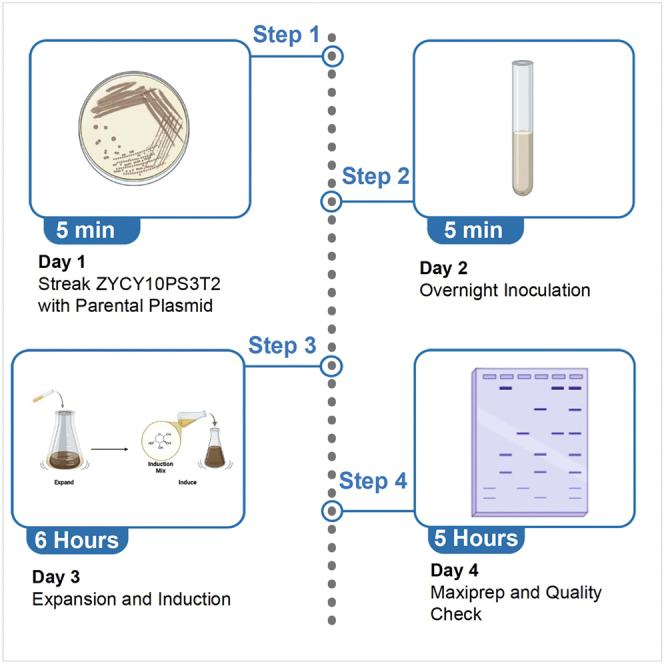

## Before you begin

The protocol describes a minicircle harvesting protocol from parental plasmids containing attB, attP, and 32 copies of Isce-I endonuclease sites in the minicircle producing bacteria, ZYCY10PS3T2.

### Background

Gene therapies offer a cure for those diseases which were uncurable in the past.^1^^,^^2^^,^^3^ Advancements of gene therapies offer to correct mutations or express the native gene for therapeutic potential. Thus, direct manipulation of the genome or replacement therapy offers immense restorative potential.^1^^,^^4^

**Figure 1:**
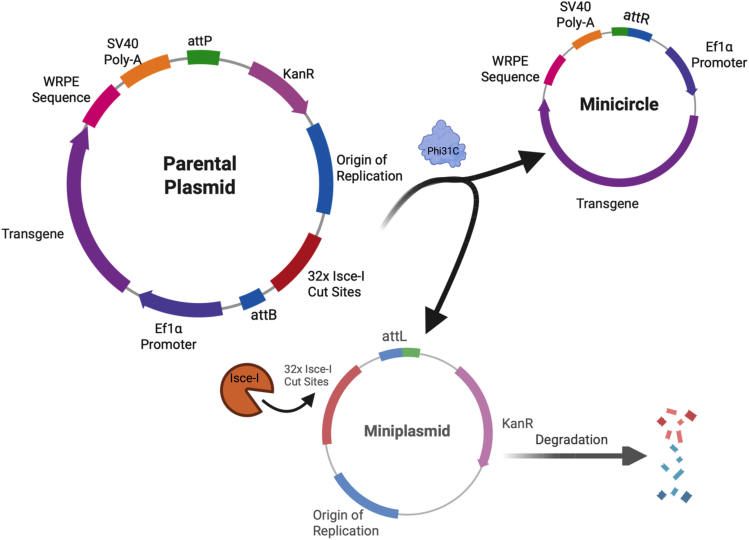
Diagram of *in vivo* recombination of the parental plasmid for minicircle creation ZYCY10P3S2T treated with arabinose induces the production of Phi31C and Isce-I. Phi31C facilitate a recombination reaction between the attP and attB sites to produce the minicircle and miniplasmid. The miniplasmid and remaining parental plasmid are subsequently degraded by the 32x Isce-I cut sites leaving the minicircle for harvesting. Figure created with Bio Render.

Non-integrating plasmids represent an attractive therapeutic option because they are easily accessible, producible and modifiable. However, prokaryotic sequences within the plasmid lend itself to transcriptional silencing, heterochromatic formation and inflammation which are not suitable for long-term expression or therapeutic use in humans.^5^^,^^6^ Minicircles are circular DNA vectors that lack bacterial or viral sequences, which result in longer transgene expression and are less immunogenic.^5^^,^^6^^,^^7^ These DNA vectors can be produced in-vivo from a specialized minicircle producer strain, ZYCY10PS3T2, which express arabinose inducible enzymes that facilitate recombination of the parental plasmid and destruction of the mini-plasmid containing the origin of replication and antibiotic resistance genes (Figure 1).^7^ However, the yield of minicircle production is low because of the need for purification steps, limiting their translational potential for gene therapies. Despite the recent improvements of minicircle production, there is still difficulty generating minicircles.^8^ Previous published procedures have produced minicircles with 96 primers for bacteria free generation using polymerase chain reaction or chromatographic gradients for purification.^9^^,^^10^ These procedures are tedious or require specialized equipment. Here we present an improved method for high yield pure minicircle production that uses up to three equivalents of an endotoxin free maxiprep kit which is cost effective compared to traditional procedures and is readily accessible in molecular biology labs. The protocol presented here differs from previously published procedures because expansion and induction are done same day. This assures that bacteria are in log phase before inducing rather than growing up an overnight culture and inducing which could result in more bacterial death. This methodology draws from the same principle from extraction kits to avoid overgrowth and genomic DNA contamination. The protocol will not cover cloning of minicircle and standard bacterial transformation but rather the harvesting from transformed ZYCY10P3S2T strains to improve minicircle yield.

### Institutional permissions

HEK 293T is considered BSL-2 specimen and NIH/3T3 is BSL-1 specimen. Take necessary precautions and wear PPE in accordance with institutional guidelines.

## Key resources table

**Table:** 

REAGENT or RESOURCE	SOURCE	IDENTIFIER
**Bacterial and virus strains**		

ZYCY10P3S2T minicircle production strain	System Biosciences	MN900A-1

**Chemicals, peptides, and recombinant proteins**		

Arabinose induction solution (20%)	System Biosciences	MN850A-1
Kanamycin monosulfate powder	Fisher Scientific	AAJ6066803
Terrific broth powder	Alfa Aesar	H26824.36
Lysogeny broth powder	Fisher Bioreagents	BP1426-2
Lysogeny broth agar powder	Fisher Bioreagents	BP1425-500
Sodium hydroxide pellets	Fisher Scientific	S318-100
Glycerol, molecular biology grade	Thermo Fisher Scientific	J61059-AP
Agarose, low melting point/molecular biology grade	Fisher Scientific	BP160-100
50x TAE	Research Products International	T60015-1000.0
SYBR Green gel stain	Invitrogen	S7563
TriTrack DNA loading dye (6X)	Fisher Scientific	Cat#FERR1161
GeneRuler 1 kb Plus DNA ladder, ready to use	Thermo Scientific	SM1333
EcorI-HF	New England Biolabs	R3101S
*Trans*IT-X2 dynamic delivery system	Mirus Bio	MIR 6000
Opti-MEM I reduced serum medium	Gibco	31985062
Dulbecco’s modified Eagle’s medium (DMEM)	ATCC	30-2002
Antibiotic-antimycotic (100X)	Gibco	15240062
Fetal bovine serum	Corning	35-015-CV
3M sodium acetate, pH 5.2	Thermo Scientific	R1181
Trypan blue solution, 0.4%	Gibco	15250061
TrypLE Express enzyme (1X), phenol red	Gibco	12605028
High-capacity cDNA reverse transcription kit	Applied Biosystems	4368814
TaqMan Fast advanced master mix for qPCR	Applied Biosystems	4444557
TempAssure 0.1 mL PCR flex-free 8-tube strips with optical cap strips	USA Scientific	1402-3600
TaqMan human TBX4 probes	Thermo Scientific	Hs01057581_m1
TaqMan mouse Tbx4 probes	Thermo Scientific	Mm01299756_g1
TaqMan mouse β-actin probes	Thermo Scientific	Mm02619580_g1
MicroAmp EnduraPlate optical 96-well clear reaction plates with barcode	Applied Biosystems	4483354
MicroAmp optical adhesive film	Applied Biosystems	4311971

**Experimental models: Cell lines**		

HEK293T	ATCC	CRL-3216
NIH/3T3	ATCC	CRL-1658

**Recombinant DNA**		

Parental plasmid MCS (empty)	System Biosciences	MN502A-1
minicircle MCS (empty)	This Study	NA
Parental plasmid mCherry-P2A-TBX4	This Study	NA
Minicircle mCherry-P2A-TBX4	This Study	NA

**Software and algorithms**		

ImageJ (FIJI)	Schindelin et al.^11^	https://imagej.net/Fiji.html#Downloads
EVOS FLAuto 2 cell imaging system	Invitrogen	https://www.thermofisher.com/us/en/home/technical-resources/software-downloads/evos-fl-auto2-imaging-system-software-download.html

**Other**		

Zyppy miniprep kit	Zymo Research	D4020
Zyppy RNA microprep kit	Zymo Research	R1051
GeneJET maxiprep endotoxin free	Thermo Fisher Scientific	K0861
Petri dish with clear lid	Fisher Scientific	FB0875712
14 mL polypropylene round-bottom tubes	Falcon	352059
24-well cell culture plate	Fisher Scientific	FB012929
Pyrex 500 mL round media storage bottle with GL45 screw cap	Corning	1395-500
Shake flask w/ 3 side and 3 bottle baffles 2,000 mL	Bellco	2543-62000
NanoDrop One microvolume UV-vis spectrophotometer	Fisher Scientific	ND-ONE-W
MaxQ floor shaker	Thermo Scientific	SHKA5000 (4348)
Sorvall LYNX 4000 centrifuge	Thermo Scientific	75006580
Fiberlite F14-6 x 250y fixed angle rotor for LYNX 4000 and 6000 superspeed centrifuges	Thermo Scientific	096-062075
Evos FL Auto 2	Invitrogen	AMAFD2000
Thermo Scientific Nalgene PPCO centrifuge bottles	Fisher Scientific	05-562-23
Magic Chef 1.3 cu. ft. 1,000 W countertop microwave oven, white	Magic Chef	https://www.tractorsupply.com/tsc/product/magic-chef-13-cf-1000w-countertop-microwave-oven-white-mcm1310w-1335994?cid=Shopping-Google-Organic_Feed-Product-1335994&srsltid=AfmBOopBnVjg2LPbGI_2OUo6wutaSgakGiA2LSTnPqIIyx8B9MQWHDTcvUk&gQT=1
C1000 Touch thermocycler	Bio-Rad	1851196
QuantStudio 3	Applied Biosystems	A28137

## Materials and equipment

**Table:** Lysogeny broth

Reagent	Final concentration	Amount
Lysogeny broth powder	N/A	6.25 g
ddH_2_O	N/A	250 mL
**Total**	N/A	**250 mL**

Store at 4°C for a month.

**CRITICAL:** Autoclave on short liquid cycle or 121°C for 15 min to sterilize before use.

**Table:** Terrific broth

Reagent	Final concentration	Amount
Terrific broth powder	N/A	12.7 g
Glycerol	N/A	1 mL
ddH_2_O	N/A	251 mL
**Total**	N/A	**251 mL**

Store at 4°C for a month.

**CRITICAL:** Autoclave on short liquid cycle or 121°C for 15 min to sterilize before use.

***Note:*** Glycerol can be difficult to pipette because of the viscosity.

**Table:** 1000x Kanamycin stock solution

Reagent	Final concentration	Amount
Kanamycin Sulfate Powder	N/A	0.5 g
ddH_2_O	N/A	10 mL
**Total**	**50 mg/mL**	**10 mL**

Store in 1000 uL aliquots in −20°C up to one year and 4°C for a month.

**CRITICAL:** Filter sterilize through a 0.22 uM filter to get rid of particulates.

**Table:** Lysogeny broth kanamycin agar plates

Reagent	Final concentration	Amount
LB Agar Powder	N/A	10 g
ddH_2_O	N/A	250 mL
1000x Kanamycin stock (50mg/mL)	50 ug/mL	250 uL
**Total**	**50 ug/mL kanamycin**	**250.250 mL**

Store at 4°C for a month.

**CRITICAL:** Autoclave on short liquid cycle or 121°C for 15 min.

**CRITICAL:** Allow the agar to cool to 55°C before adding antibiotic or it will be degraded.

**CRITICAL:** Allow plates to rest at 25°C–27°C for 16 h before use to allow drying.

**Table:** 1M NaOH solution for induction

Reagent	Final concentration	Amount
NaOH Pellets	N/A	1 g
ddH_2_O	N/A	25 mL
**Total**	**1M**	**25 mL**

Store at 25°C for a year.

**CRITICAL:** NaOH is caustic and can cause injury if not handled properly. Assure that proper PPE is worn with extra precautions on weighing and dissolving the pellets.

***Note:*** Dissolve the NaOH in 20 mL first then bring up to volume.

## Step-by-step method details

### Day 1: Instructions for streaking of ZYCY10P3S2T with parental plasmid

**Timing: 5 min**

This section of the protocol covers single colony inoculation to grow a starter culture for expansion.

1. Using the agar plate with antibiotic, streak ZYCY10P3S2T with parental plasmid using a dilution streak technique to isolate single colonies.
  a. Grow for 12–16 h at 37°C.
  b. Store plate in 4°C for up to a week.

### Day 2: Instructions for inoculation of ZYCY10P3S2T with parental plasmid

**Timing: 5 min**

This section of the protocol covers how to inoculate colonies and grow bacteria to reach sufficient OD (10–11) for expansion into a larger culture.

2. Add 3 mL of terrific broth kanamycin to a 14 mL polypropylene round-bottom tube.
  a. Inoculate a single colony into the tube.
  b. Repeat so you have duplicate tubes.
  c. Incubate at 30°C and shaking at 250 RPM using the MaxQ floor shaker.
  d. Begin culture 5–6 pm and come back at 9 am to take out cultures.

***Note:*** If the OD overshoots the 10–11 range, it will still be usable. In our experience, ODs above 11 take longer to get out of lag phase.

**CRITICAL:** Make sure to maintain 1:5 culture to air volume for growth to assure proper oxygenation. Take out the cultures after 16 h MAX to assure for healthy growth downstream.

**CRITICAL:** Do not use lysogeny broth for this step because terrific broth is buffered mitigating the pH effects of growth byproducts and can reach the higher OD required for expansion the following day.

***Note:*** Inoculate in duplicates in case one is not successful.

### Day 3: Instructions for regrowth and induction of ZYCY10P3S2T with parental plasmid

**Timing: 6 h**

This section of the protocol describes the expansion of the starter culture and induction for minicircle production.

3. Dilution of the cultures into a bigger culture for regrowth into a 1000 mL baffled flask with 201 mL TB Kanamycin.
  a. Measure the OD of the cultures to determine the values for proper dilution using the Nanodrop one OD setting.
    ***Note:*** 2 uL of blank and culture should be sufficient to get an accurate reading.
  b. Add 201 mL of terrific broth kanamycin into the 1000 mL baffled flask.
  c. Dilute the starter culture such that the starting OD is 0.1.
  d. Regrow the bacteria at 37°C and 250 RPM shaking until the OD is around 4 which will take around 3–4 h depending on the health of the bacteria.
    ***Note:*** Measure the OD every hour, then every 30 min once it enters exponential growth.
    ***Note:*** Regrow at 37°C and not 30°C for faster growth before induction.
4. Once OD of 4 is reached, place the bacteria on the benchtop during the creation of the induction mix.
  a. Shift the temperature of the shaker to 30°C to allow for cooling for subsequent induction step.
  b. Collect 1 mL of bacteria for miniprep for pre-induction culture.
  c. Pellet the cells at 15000 × g at 4°C for 30 seconds.
  d. Aspirate media without disrupting the pellet.
  e. Store at −80°C.
  f. Take 100 mL of the bacteria and add to another 1000 mL baffled flask. There should now be two 1000 mL baffled flasks each with 100 mL of bacteria.
5. Prepare the induction mix (200 mL) in the order shown in an empty, separate flask. (Table 1).
  a. Gently swirl the flask for at least 10 seconds to assure homogenous solution.

**Table 1:** Induction mix

Reagent	Final concentration	Amount
Lysogeny Broth with NO ANTIBIOTIC	N/A	196 mL
1M NaOH	20 mM	4 mL
20% Arabinose	0.02%	200 uL
Total	N/A	200.2 mL

***Note:*** The induction mix should be 25°C−27°C prior to addition. This will help cool the bacteria to reach 30°C.

**CRITICAL:** Always make fresh and do not store. There are no antibiotics thus stored induction media is susceptible to contamination.

6. Add 100 mL of induction mix per flask.
  a. Incubate at 30°C shaking 250RPM for 90 min.

***Note:*** The final volume will be 200 mL of bacteria and induction mix to maintain the 1:5 culture to air volume for optimal oxygenation.

**CRITICAL:** Phi31C Integrase functions optimally at 30°C which is required for splicing of attB and attP sites for minicircle creation. However, if the shaker cannot maintain 30°C, 32°C is also acceptable.

7. After 90 min of incubation at 30°C, shift the temperature of the shaker to 37°C.
  a. Incubate for 60 min with shaking at 250RPM.

**CRITICAL:** Isce-I endonuclease functions optimally at 37°C which is essential for degradation of the miniplasmid and leftover parental plasmids.

8. Collect 1 mL from each flask for induction quality check using miniprep in a microcentrifuge tube.
  a. Pellet the cells at 15000 × g for 30 seconds.
  b. Remove media from the cells without disrupting the pellet.
  c. Store at −80°C
9. Record the final OD using the nanodrop one and volume of culture.

**CRITICAL:** This will be used for maxiprep calculations downstream otherwise the biomass might exceed what a standard reaction which could lead incomplete lysis and thus contamination.

10. Using the 250 mL ultracentrifuge bottles, spin down the two induction cultures separately.
  a. 5000 × g at 4°C for 10 min.
11. Remove the supernatant without disrupting the pellet.
  a. Wash the pellet once with 4°C 50 mL of ultrapure water without resuspending the pellet.
    **CRITICAL:** Wash with water and not buffer. The water washes the residual terrific broth which could affect subsequent lysis steps downstream.
  b. Remove water without disturbing the pellet.
  c. Store in −80°C for 12–16 h until the next day.
    ***Note:*** The −80°C freeze thaw helps with lysis the following day.
    **Pause point:** The bacteria can be stored in the −80°C for a week if lysis cannot be completed the following day.

### Day 4: Instructions for DNA extraction of minicircle using mini- and maxiprep

**Timing: 6 h**

This section of the protocol describes the how to miniprep, check quality of the minicircle production and maxiprep.

12. Take out the miniprep cultures and let thaw on ice.
13. Using the Zyppy Miniprep Kit, resuspend the pellet in 600 uL of ultrapure water.
  a. Proceed with manufacturer’s protocols. LINK.
  b. Nanodrop the prepped parental and minicircle DNA to get DNA concentration for restriction digest step.

***Note:*** To increase yield, incubate the elution buffer at 55°C–60°C before elution.

14. Perform a restriction digest on the mini-prepped pre- and post-induction pellets (Table 2).
  ***Note:*** X refers to the amount of volume required to aliquot 250 ng of DNA. This will be subtracted to get the amount of water needed to bring the reaction up to 20 uL.
  a. Incubate the reaction at 37°C for 30 min.
    ***Note:*** Use a restriction enzyme that is present in both the parental and minicircle to linearize and run on an agarose gel. For our minicircles, the enzyme of choice was ECORI-HF.

**Table 2:** Restriction mix

Reagent	Final concentration or amount	Amount
Parental or minicircle DNA	250 ng	x uL
Ultrapure Water	N/A	17 – x uL
10x rCutsmart Buffer	1x	2 uL
EcorI-HF	20 units	1 uL
Total	N/A	20 uL
15. Cast a 1% agarose 1x TAE gel.
  a. Weigh out 1 g of melting point agarose in a 500 mL round media pyrex storage bottle.
  b. Add 100 mL of 1x TAE.
  c. Using a microwave, heat the TAE agarose for 30 seconds, pause and release pressure every 15 seconds.
  d. Once the agarose is completely dissolved, run the bottle under cool water for 30 seconds.
  e. Add 5 uL of the Sybr Green gel stain and solidify for 45 min on benchtop.
16. Add 4 uL of 6x Loading dye.
  a. Add 5 uL of 1 kb ladder to the first lane then load corresponding samples.
  b. Run the gel at 100V for 30 min.
17. Visualize using a gel doc.

***Note:*** After electrophoresis, if there is less than <10% parental plasmid contamination, it will be acceptable for experiments.

***Note:*** However, genomic DNA contamination signifies issues with sample health or miniprep extraction. If the minicircle appears acceptable, proceed to maxi prep.

***Note:*** See Figure 2 for examples of acceptable vs unacceptable minicircles.

***Note:*** Additionally, if your lab does not use the following maxiprep kit, this could be substituted for the kit of choice.

**CRITICAL:** Assure that the substituted kit removes endotoxin before proceeding with in-vivo or in-vitro studies.

18. Based on the gel electrophoresis of the parental plasmid and minicircles, continue to maxiprep. Scale the resuspension, lysis, neutralization buffers, endotoxin binding and isopropanol steps before column binding. (Table 3) LINK to online protocol.
  a. Example Calculation: Scaling for OD 6 in 200 mL of post-induction reaction.
  b. Write out the formula for scaling calculation. Maximum Culture Volume (mL) = 750/OD600.
  c. Divide by the OD. Maximum Culture Volume (mL) = 750/6.
  d. The result from 18c will be the maximum amount of culture volume for this optical density. Maximum Culture Volume (mL) = 125.
  e. Divide the final volume of culture by the calculated volume. This will be your scaling factor. Scaling Factor = 200 mL/125 mL which is equal to 1.6.

**Table 3:** Maxiprep scaling example

Reagent	Initial amount from protocol	Scaled amount (1.6x)
Resuspension Solution with RNase A	7 mL	11.2 mL
Lysis Solution	7 mL	11.2 mL
Neutralization Solution	7 mL	11.2 mL
Endotoxin Binding Reagent	1 mL	1.6 mL
Isopropanol	4 mL	6.4 mL

***Note:*** From the calculated scaling factor, to properly lyse all the bacteria, the reagents must be scaled from steps 1–6 by 1.6x of the maxi kit protocol.

**CRITICAL:** For each 20 mL of flow through from step 8, 8 mL of isopropanol must be added to allow for proper DNA binding for column purification in subsequent steps.

**CRITICAL:** Use one white column per flask and combine the flow throughs from both white columns onto one blue column.

***Note:*** To increase elution yield, incubate the elution buffer in 55°C–60°C at the beginning of the extraction.

19. After scaling calculation, follow the manufacturer’s protocols for the GeneJet Endo-free Plasmid Maxiprep Kit (K0861). Refer to step 18 for link to online protocol.
  a. Prepare a digest and gel like in steps 16 and 17 to assess quality.

**Figure 2:**
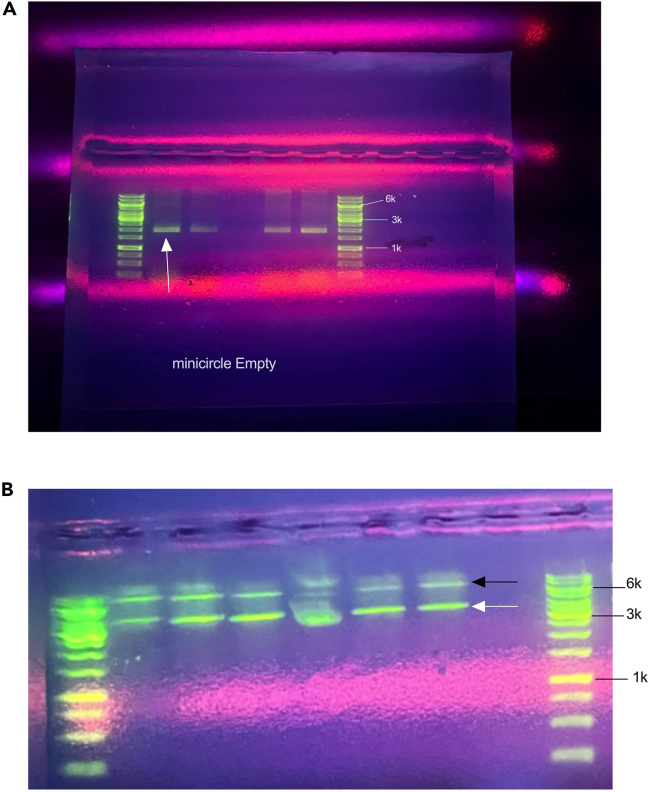
Examples of acceptable and unacceptable minicircles post-induction (A) demonstrates four elutions of acceptable empty minicircles made from this protocol. (B) demonstrates high parental plasmid (PP) contamination alongside with minicircle production that would not be amenable for downstream applications. The black arrow points to left over parental plasmid and the white arrow points to the minicircle.

***Note:*** No genomic DNA and parental plasmid less than 10% should be present to be deemed acceptable, store in −20°C indefinitely and use for in-vivo or in-vitro assays (Figures 2 and S1).

***Note:*** An acceptable 260/280 ratio is between 1.8–2.

***Note:*** An acceptable 260/230 ratio is between 2–2.7.

**CRITICAL:** Follow the lysis timing exactly as described. Over lysis will release genomic DNA and contaminate your preparation.

### Instructions for transfection of minicircle mCherry-P2A-TBX4 for fluorescence visualization

**Timing: up to 7 days**

The following step is optional because the minicircle used in the protocol contained a fluorescent reporter but provides additional information in a human cell line to determine if the minicircle DNA that was extracted was minicircle and not miniplasmid. However, other biochemical techniques can be used to detect the expressed transgene such as western blot, qPCR or immunocytochemistry.

***Note:*** Any method to introduce plasmids into cells should work. We prefer the TransIT-X2 system, but this should work with calcium phosphate or electroporation. However, other transfection protocols were not tested so transfection efficiency may vary.

20. Thaw HEK293T cells in hands by gripping them for 2 min. After, the following steps will be in the hood aside from the centrifugation steps.
  a. Resuspend the cells in a 50 mL conical with 10 mL of DMEM 10% FBS 1% Antianti warmed to 37°C.
  b. Pellet the cells at 300 g for 5 min at 4°C and remove the media.
  c. Resuspend the cells in 10 mL of DMEM 10% FBS 1% Antianti and pellet again at the same speed.
  d. Remove media while leaving the pellet undisturbed and resuspend in 10 mL of fresh media.
  e. Plate onto a 10 cm plate and leave in the incubator set at 37°C and 5% CO_2_.
  f. When the cells reach around ∼80–90% confluence. Proceed to split.
  g. Aspirate all media in the dish and add 5 mL of TrypLE.
  h. Incubate at 37°C for 5 min.
  i. Wash the plate with 5 mL of DMEM 10% FBS 1% Antianti and collect contents into a 50 mL conical.
  j. Pellet the cells at 700 g for 5 min.
  k. Remove the media and resuspend in 5 mL of DMEM 10% FBS 1% Antianti.
  l. To a new 10 cm plate, add 9 mL of DMEM 10% FBS 1% Antianti and add 1 mL of the cell suspension to the plate.
  m. Repeat twice to ensure healthy growth before transfection.
21. When cells are passaged at least twice, seed for transfection.
  a. To the 10 cm plate that is around 80% confluent, remove all the media, and add 5 mL of TrypLE.
  b. Incubate at 37°C for 5 min.
  c. Add 5 mL of cell culture media, wash the plate and collect into a 50 mL conical.
  d. Spin the cells at 700 g for 5 min at 4°C.
  e. Remove the media and resuspend the cell pellet in 5 mL of warmed media.
  f. To a separate Eppendorf tube, add 80 uL of Trypan blue with 20 uL of the cell mixture.
  g. Add 10 uL of the hemacytometer to count the cells.
  h. Following cell counts, dilute the cells to 150,000 cells/mL in fresh media.
  i. Add 2 mL of the diluted media to the desired wells that are to be transfected.
  j. Incubate in the 37°C incubator for 12–16 h.
22. To prepare for transfection, allow TransIT-X2 and OptiMEM to equilibrate to room temperature before use.
  a. Prepare the transfection mixture exactly from top to bottom and mix well between addition of reagents making sure that none of it gets onto the sides. (Table 4).
    **CRITICAL:** Add the TransIT-X2 last. This transfection reagent forms complexes as soon as it is added to the OptiMEM. If not added last, transfection complexes will be formed without your minicircle.
    ***Note:*** X refers to the amount of volume required to aliquot 2500 ng of DNA. This will be subtracted to get the amount of OptiMEM needed to bring the reaction up to 257.5 uL.

**Table 4:** Transfection mix per one well of a 6-well plate

Reagent	Final concentration or amount	Amount
Minicircle DNA	2500 ng	x uL
OptiMEM	N/A	250 – x uL
TransIT-X2	N/A	7.5 uL
Total	N/A	257.5 uL
  b. Pipette up and down after the addition of TransIT-X2 and allow to incubate for 30 min in the hood.
  c. During the incubation step aspirate the media of the wells to be transfected and add 2.5 mL of warmed media.
  d. After incubation, add the minicircle transfection mixture dropwise.
  e. Gently rock the plate in vertically and horizontally to evenly distribute the mixture and put cells back into the incubator to incubate for 12–16 h.
  f. The following day, remove the media and add 3 mL of warmed media.
  g. Image using a fluorescent microscope 48 h post-transfection to visualize minicircle.

### Instructions for transfection of minicircle mCherry-P2A-TBX4 for transcription production

**Timing: up to 7 days**

This section describes transfection, RNA extraction, reverse transcription and quantitative PCR to test the transcript production of minicircle TBX4 in a murine cell line.

***Note:*** Any method to introduce plasmids into cells should work. We prefer the TransIT-X2 system, but this should work with calcium phosphate or electroporation. However, other transfection protocols were not tested so efficiency may vary.

23. Thaw NIH/3T3 cells in hands by gripping them for 2 min. After, the following steps will be in the hood aside from the centrifugation steps.
  a. Resuspend the cells in a 50 mL conical with 10 mL of DMEM 10% FBS 1% Antianti warmed to 37°C.
  b. Pellet the cells at 300 g for 5 min at 4°C and remove the media.
  c. Resuspend the cells in 10 mL of DMEM 10% FBS 1% Antianti and pellet again at the same speed.
  d. Remove media while leaving the pellet undisturbed and resuspend in 10 mL of fresh media.
  e. Plate onto a 10 cm plate and leave in the incubator set at 37°C and 5% CO_2_.
  f. When the cells reach around ∼80–90% confluence. Proceed to split.
  g. Aspirate all media in the dish and add 5 mL of TrypLE.
  h. Incubate at 37°C for 5 min.
  i. Wash the plate with 5 mL of DMEM 10% FBS 1% Antianti and collect contents into a 50 mL conical.
  j. Pellet the cells at 700 g for 5 min at 4°C.
  k. Remove the media and resuspend in 5 mL of DMEM 10% FBS 1% Antianti.
  l. To a new 10 cm plate, add 9 mL of DMEM 10% FBS 1% Antianti and add 1 mL of the cell suspension to the plate.
  m. Repeat twice to ensure healthy growth before transfection.
24. When cells are passaged at least twice, seed for transfection.
  a. To the 10 cm plate that is around 80% confluent, remove all the media, and add 5 mL of TrypLE.
  b. Incubate at 37°C for 5 min.
  c. Add 5 mL of cell culture media, wash the plate and collect into a 50 mL conical.
  d. Spin the cells at 700 g for 5 min at 4°C.
  e. Remove the media and resuspend the cell pellet in 5 mL of warmed media.
  f. To a separate Eppendorf tube, add 80 uL of Trypan blue with 20 uL of the cell mixture.
  g. Add 10 uL of the hemacytometer to count the cells.
  h. Following cell counts, dilute the cells to 150,000 cells/mL in fresh media.
  i. Add 2 mL of the diluted media to the desired wells that are to be transfected.
  j. Incubate in the 37°C incubator for 12–16 h.
25. To prepare for transfection, allow TransIT-X2 and OptiMEM to equilibrate to room temperature before use.
  a. Prepare the transfection mixture exactly from top to bottom and mix well between addition of reagents making sure that none of it gets onto the sides.
    **CRITICAL:** Add the TransIT-X2 last. This transfection reagent forms complexes as soon as it is added to the OptiMEM. If not added last, transfection complexes will be formed without your minicircle.
  b. Make one transfection mix for mcTBX4 and mcEmpty. (Table 5).
    ***Note:*** X refers to the amount of volume required to aliquot 2500 ng of DNA. This will be subtracted to get the amount of OptiMEM needed to bring the reaction up to 257.5 uL.

**Table 5:** Transfection mix per one well of a 6-well plate

Reagent	Final concentration or amount	Amount
Minicircle DNA	2500 ng	x uL
OptiMEM	N/A	250 – x uL
TransIT-X2	N/A	7.5 uL
Total	N/A	257.5 uL
  c. Pipette up and down after the addition of TransIT-X2 and allow to incubate for 30 min in the hood.
  d. During the incubation step aspirate the media of the wells to be transfected and add 2.5 mL of warmed media.
  e. After incubation, add the minicircle transfection mixture dropwise.
  f. Gently rock the plate in vertically and horizontally to evenly distribute the mixture and put cells back into the incubator to incubate for 12–16 h.
  g. The following day, remove the media and add 3 mL of warmed media.
26. After 72 h post-transfection, prepare for RNA extraction.
  a. Aspirate all media from the wells.
  b. Add 500 uL of TryPLE and incubate at 37°C for 3 min.
  c. After, wash the plate with 1 mL of DMEM 10% FBS 1% Antianti.
  d. Collect the contents into a 1.5 mL tube and spin the cells 500 g for 5 min at 4°C.
  e. Leaving the cell pellet untouched, aspirate 1450 uL (725 uL twice) using a pipette.
  f. Add 300 uL of RNA lysis buffer from the RNA Microprep kit and follow manufacturer’s instructions for the rest of the extraction. LINK.
  g. Elute in 15 uL of ultrapure water and put on ice to maintain RNA integrity.
    **Pause point:** RNA is stable at −80°C for 1 month without nuclease contamination. Make sure to nanodrop again after thawing to check concentration.
27. Measure the RNA concentration using the nanodrop.
  **CRITICAL:** Assure the A260/A280 ratio is above 2. Otherwise, there may be DNA that could influence the qPCR results.
  a. Using the Nano dropped RNA concentrations, add 500 ng of RNA into a final volume of 10 uL.
    **CRITICAL:** Use RNase/DNase free water bring this up to volume otherwise downstream results could be negatively affected by RNase presence.
    ***Note:*** If the amount of volume required for 500 ng is higher than 10 ul, subtract the amount of water needed to add into the cDNA synthesis step in the next step.
  b. Assemble the 2x cDNA synthesis mix on ice in a new 1.5 mL tube as follows adding reagents from the top of the list to bottom (Table 6).
    **CRITICAL:** Make sure to multiply by number of reactions to get appropriate volumes:
    ***Note:*** Thaw reagents on ice.

**Table 6:** Reverse transcription mix for one reaction

Reagent	Amount
10x RT Buffer	2 uL
25x dNTP Mix	0.8 uL
10x RT Random Primers	2 uL
RNse/DNse Free Water	4.2 uL
MultiScribe Reverse Transciptase	1 uL
Total	10 uL
  c. After assembly of the master mix, grab an 8-strip PCR tube an aliquot 10 uL of the master mix for the samples to be reverse transcribed.
  d. Add 10 uL of the 500 ng RNA mix, from 5a, and program the thermocycler with the conditions in Table 7.
    **Pause point:** cDNA is stable at −20°C for years without nuclease contamination.

**Table 7:** Thermocycler conditions for reverse transcription

Step	1	2	3	4
Temperature	25°C	37°C	85°C	4°C
Time	10 minuets	120 minuets	5 minuets	Indefinite
28. After cDNA synthesis, proceed to qPCR set up.
  a. Dilute the cDNA 1/10. Add 3 uL of undiluted cDNA and 27 uL of nuclease free water.
    ***Note:*** This will be a sufficient volume for three probes in triplicate and account for volume loss during transfers.
    ***Note:*** The amount of RNA is 500 ng in 20 uL of reaction mix resulting in a concentration of 2.5 ng/uL. In our hands, the optimal amount of cDNA per reaction is 5 ng.
  b. Assemble the Taqman reaction mix per probe. The final volume is 10 uL with cDNA added per well (Table 8).
    ***Note:*** To cover for volume loss during transfer, make two additional reactions.

**Table 8:** qPCR Taqman master mix

Reagent	Amount
2x Taqman Fast Advanced Master Mix	5 uL
20x Taqman qPCR Probes	0.5 uL
RNse/DNse Free Water	2.5 uL
Total	8 uL
  c. Using the 96 well qPCR plate, pipette 2 uL of cDNA per desired well.
  d. Tap the plate on the bench three times to move the liquids to the bottom of the wells then add 8 uL of reaction mix to each well.
  e. Tap the plate three times on bench.
  f. Add the optical adhesive seal.
  g. Put the plate into the Quant studio 3 and select Comparative CT.
    ***Note:*** Be careful to not select Comparative CT with melt. This protocol is for Sybr-green and NOT taqman.
  h. Analyze the data using the ddCT method.

## Expected outcomes

After inoculation of the starter culture in terrific broth kanamycin media, an appropriate OD will be around 10–11. The higher density is acceptable because this culture will be re-inoculated and expand for regrowth in a larger culture. The regrown cultures typically enter log phase after 3–4 h of lag (Table 9).

**Table 9:** OD measurements during regrowth of larger culture for parental plasmid mCherry-P2A-TBX4 before induction

Time (h)	OD
0	0.1
1	0.12
2	0.30
3	0.98
3.5	1.8
4.0	3.7

**Figure 3:**
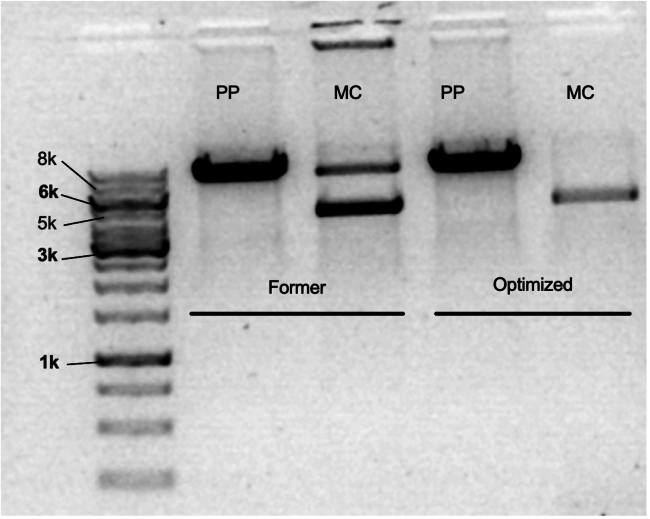
Gel electrophoresis comparing old and optimized induction procedures An inverted gel comparing previous established procedures and our optimized method using minipreps and restriction digest with a clear lack of parental plasmid in the optimized elution. The lane all the way to the left is the ladder with corresponding kb sizes. Bolded numbers refers to the bolded base sizes. Former refers to old protocol the optimized one was based on. Optimized is the protocol presented in this procedures manuscript. PP = Parental plasmid. MC = Minicircle.

**Figure 4:**
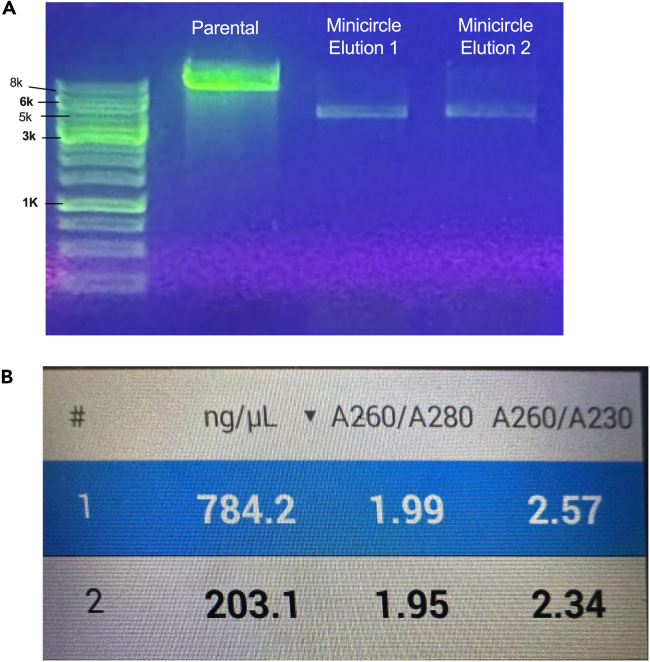
Gel electrophoresis of induction of mCherry-P2A- TBX4 minicircles with corresponding nanodrops Gel showing parental plasmid and two separate elutions of minicircle post-induction of mCherry-P2A-TBX4 showing clean productions. (A) shows gel electrophoresis of DNA ladder (Ladder), Parental Plasmid (PP) from preinduction miniprep and two separate elutions of minicircle (MC) from the GeneJet Maxiprep which correspond to minicircle 1 (MC1) and minicircle 2 (MC2). The minicircles are lower in size which correspond to successful induction and lack parental plasmid band noted demonstrating lack of contamination. (B) shows nanodrop quantifications from the two minicircle elutions. The minicircle elution corresponds the number on the nanodrop.

Compared to previous procedures, we find our method produces cleaner minicircle eliminating purification steps that reduce yield (Figures 3 and S2). Previously published protocols expand overnight then induce which could introduce contaminant DNA due to overgrowth.^7^ Following the printed clean up steps from the manufacturer, our total yields were low (Table 10). However, with our new protocol, the total yields can be up to 20-fold higher compared to the old protocol (Figures 4 and S3) (Table 10). This may be in part because culture expansion is conducted on the same day as the induction. Typical minipreps of the pre and post induction are expected to be low. In our hands, our yields range from 30–50 ng/uL eluted in 25 uL of buffer which is fine because you are looking for quality of the induction. Table 10 shows post-induction volumes, ODs, and micrograms of various minicircle maxi-extracted using this optimized protocol.

**Table 10:** Pre- and post-induction of the old minicircle protocol with cleanup steps required to remove genomic and parental contaminating DNA

	Pre-induction volume	Post-induction volume	Final OD	Total ug post-clean up
**Old protocol minicircle**				

TBX4	200 mL	600 mL	7	57.3
Empty	200 mL	400 mL	5.2	43.4

**Optimized protocol minicircle**				

mCherry-P2A-TBX4	200 mL	400 mL	6.7	780
Empty 1	200 mL	400 mL	7	970.4
Empty 2	200 mL	400 mL	6.3	786.4
FOXM1	200 mL	400 mL	8	907.2
mScarlet3-P2A-FOXM1	100 mL	200 mL	9	440.8

**Figure 5:**
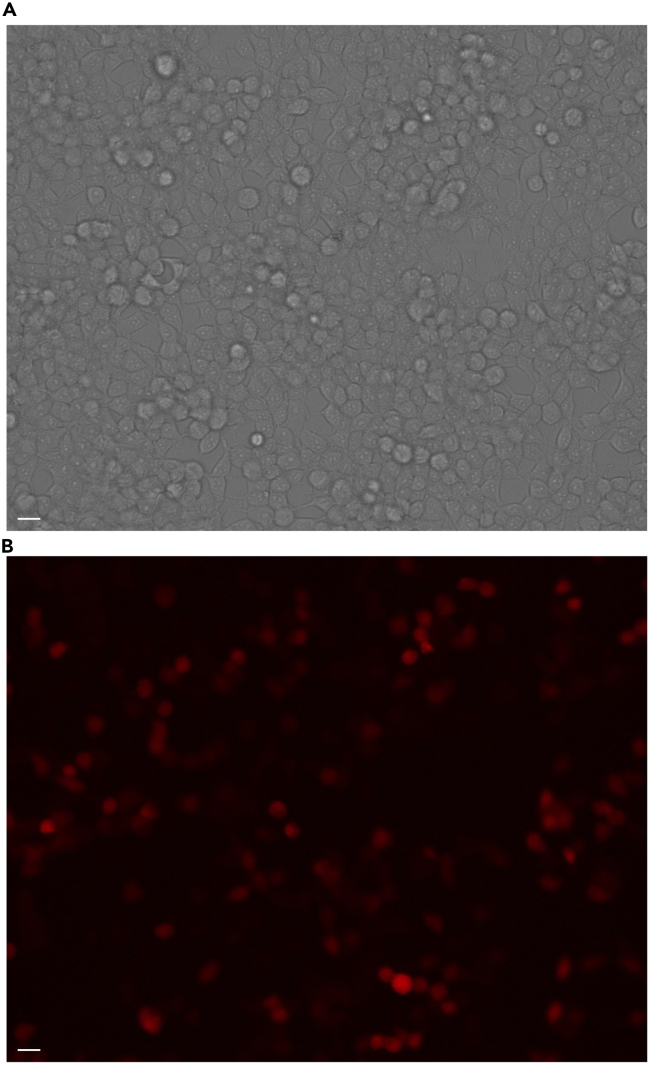
Transfection of mCherry minicircles into HEK293T cells HEK293T cells were transfected with mCherry minicircle and incubated for 48 h before visualization using the RFP filter set. (A) is the brightfield image and (B) demonstrates the red fluorescence from the mCherry minicircles. Scale bar = 20 um.

**Figure 6:**
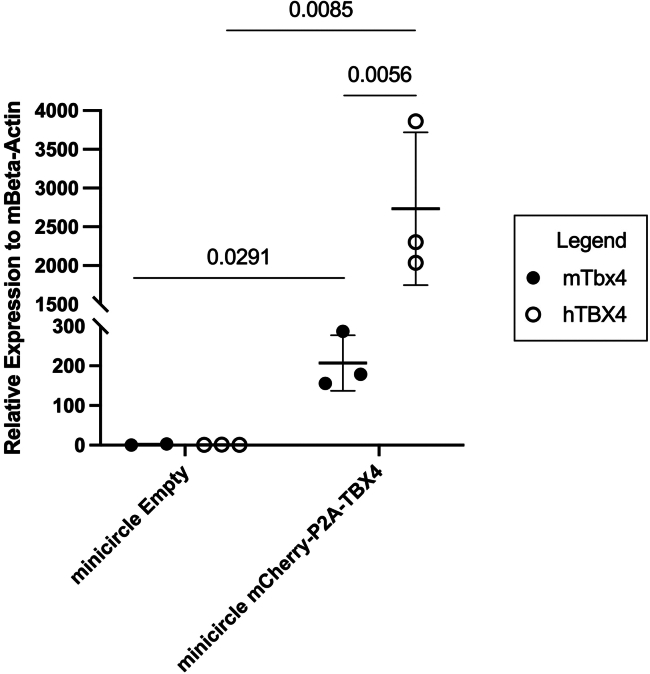
qPCR results comparing mouse and human expression of TBX4 in NIH/3T3 cells NIH/3T3 cells were transfected with either Empty or mCherry-P2A-TBX4 minicircles and incubated for 72 h before RNA extraction and reverse transcription. Mouse and human TBX4 was analyzed in both Empty and mCherry-P2A-TBX4 minicircles compared to mouse beta-actin expression. Values above the line are p-values between compared conditions. Data are represented as mean ± SD. *p*-values were calculated using a two-tailed T-test. *p* < 0.05 was deemed significant.

After 24 h, the mCherry fluorescent signal from the minicircle should be visible using conventional fluorescence microscopy (Figure 5).^12^^,^^13^^,^^14^ However, if the produced minicircle contains no fluorescent markers, it is necessary to check with other biochemical detection techniques like western blot or qPCR before proceeding to in-vivo gene therapy applications.^15^^,^^16^^,^^17^^,^^18^^,^^19^^,^^20^ Specifically, nanoparticle systems for selective organ nucleic acid delivery.^21^^,^^22^^,^^23^^,^^24^^,^^25^ As alternatives to mCherry, other reporters can be used, such as green fluorescent protein (GFP), β-galactosidase, or luciferase.^26^^,^^27^^,^^28^^,^^29^^,^^30^ For transcriptional analysis, we find that transcript production in-vitro is optimally measured after 72 h of post-transfection producing at least 2000x fold expression compared to beta-actin in a murine cell line, demonstrating versatility of minicircles in both mouse and human models (Figure 6).

## Limitations

As with all DNA extraction protocols, the user must be proficient at extraction. Care must be taken to follow the lysis and neutralization steps to prevent denaturation of supercoiled minicircle DNA and introduction of genomic nucleic acid content. Additionally, it is important to assure the parental plasmid being used for induction contains an attP and attB site to ensure proper splicing of the minicircle. This protocol is only optimized for ZYCY10PS3T E. coli and no other strains as they do not contain the necessary machinery to synthesize the required enzymes. Additionally, the parental plasmid and miniplasmid must contain IsceI endonuclease sites to facilitate degradation. It is important to note how fast the floor shaker can change and maintain temperatures. If the floor shaker cannot change temperature quick enough, then the incubation temperatures may need to be adjusted to be accounted.

## Troubleshooting

### Problem 1

Singular band present above 10 kb that is unchanged after restriction digest. Day 4: Extraction of minicircles using mini and maxiprep, step 17.

### Potential solution

• If this is the only band present with no parental or minicircle present, this suggests that there is no parental plasmid in the minicircle producer strain or that the bacteria lost the plasmid during growth.
• Assure that antibiotics are added to the agar plate and present in the terrific broth during growth and expansion the subsequent day.
• Check to make sure the parental plasmid is intact or is transformed into the bacteria by miniprep and sequencing before starting the induction process.

### Problem 2

Slow bacterial growth upon expansion into larger culture. Day 3: Regrowth and induction of ZYCY10P3S2T with parental plasmid, step 3.

### Potential solution

If the culture does not enter log phase after 5 h of expansion, restart by picking a new colony.

### Problem 3

Genomic DNA contamination. Day 4: DNA extraction of minicircle using mini and maxiprep, step 17 and 19.

### Potential solution

• Stay on the lower end of the lysis steps. If the protocol says to gently invert 5–7 times, then invert 5 times gently. Additionally, check the integrity of the lysis reagent to make sure these are tightly sealed. Evaporation of the lysis reagent could potentially increase the concentration and thus release genomic DNA.
• Additionally, if this specific prep is needed and there is no option to restart, treatment with Exonuclease V (RecBCD) Cat number M0345L followed by DNA purification to remove proteins and nucleotide fragments. We recommend NucleoSpin Gel and PCR Clean-up Maxi cat number 740610.20 from Takara bio. M0345L followed by DNA purification to remove proteins and nucleotide fragments.

### Problem 4

More than >10% parental plasmid after induction of minicircle. Day 4: DNA extraction of minicircle using mini and maxiprep, step 17 and 19.

### Potential solution

• Check to assure the floor shaker is reaching appropriate temperature. Include a thermometer inside the floor shaker to monitor temperature.
• Increase the duration of 30∗C to 120 min instead of 90 min. Additionally, increase the incubation of 37∗C to 90 min instead of 60 min. This will give more time for the Isce-I endonuclease to degrade the unspliced parental plasmid.
• Additionally, if this specific prep is needed and there is no option to restart, find a restriction enzyme that is present in the parental plasmid but NOT the minicircle, then do a DNA purification to remove the proteins and nucleotide. We recommend NucleoSpin Gel and PCR Clean-up Maxi cat number 740610.20 from Takara bio.

### Problem 5

Lack of growth in the starter culture. Day 2: Inoculation of ZYCY10P3S2T with parental plasmid, step 2.

### Potential solution

• Check to make sure you are adding the correct antibiotic for the parental plasmid.

Additionally, check to make sure the terrific broth was made correctly because of the addition of glycerol.

## Resource availability

### Lead contact

Further information and requests for resources and reagents should be directed to and will be fulfilled by lead contact, Vladimir V. Kalinichenko (vkalin@arizona.edu).

### Technical contact

Technical questions on executing this protocol should be directed to and will be answered by the technical contact, Jonathan Do (jondo@arizona.edu).

### Materials availability

There are restrictions on the availability of parental plasmid mCherry-P2A-TBX4 and minicircle mCherry-P2A-TBX4 due to a concurrent study being conducted.

### Data and code availability

This protocol generated no new data or code.

## Acknowledgments

This work was supported by 10.13039/100000050National Heart, Lung, and Blood Institute grants R01 HL141174 (V.V.K.), R01 HL149631 (V.V.K.), and R01 HL152973 (V.V.K. and T.V.K.). The authors acknowledge Fatemeh Kohram, who provided the empty parental plasmid and mCherry-P2A-Foxf1 plasmid for TBX4 subcloning.

## Author contributions

Conceptualization, J.D.; writing – original draft, J.D.; writing – reviewing and editing, J.D., Z.D., and V.V.K.; methodology, J.D., G.V., Y.M., and M.A.; funding acquisition, V.V.K.; study supervision, V.V.K. and T.V.K.

## Declaration of interests

The authors declare no competing interests.
