# Supplementary material for: Protocol for minicircle production for gene therapy without subsequent cleanup steps
Source: STAR Protoc. 2025 Jul 24;6(3):103982. doi: 10.1016/j.xpro.2025.103982 (PMC12311595; doi:10.1016/j.xpro.2025.103982)
Supplement: Document S1. Figures S1–S3 [file mmc1.pdf]

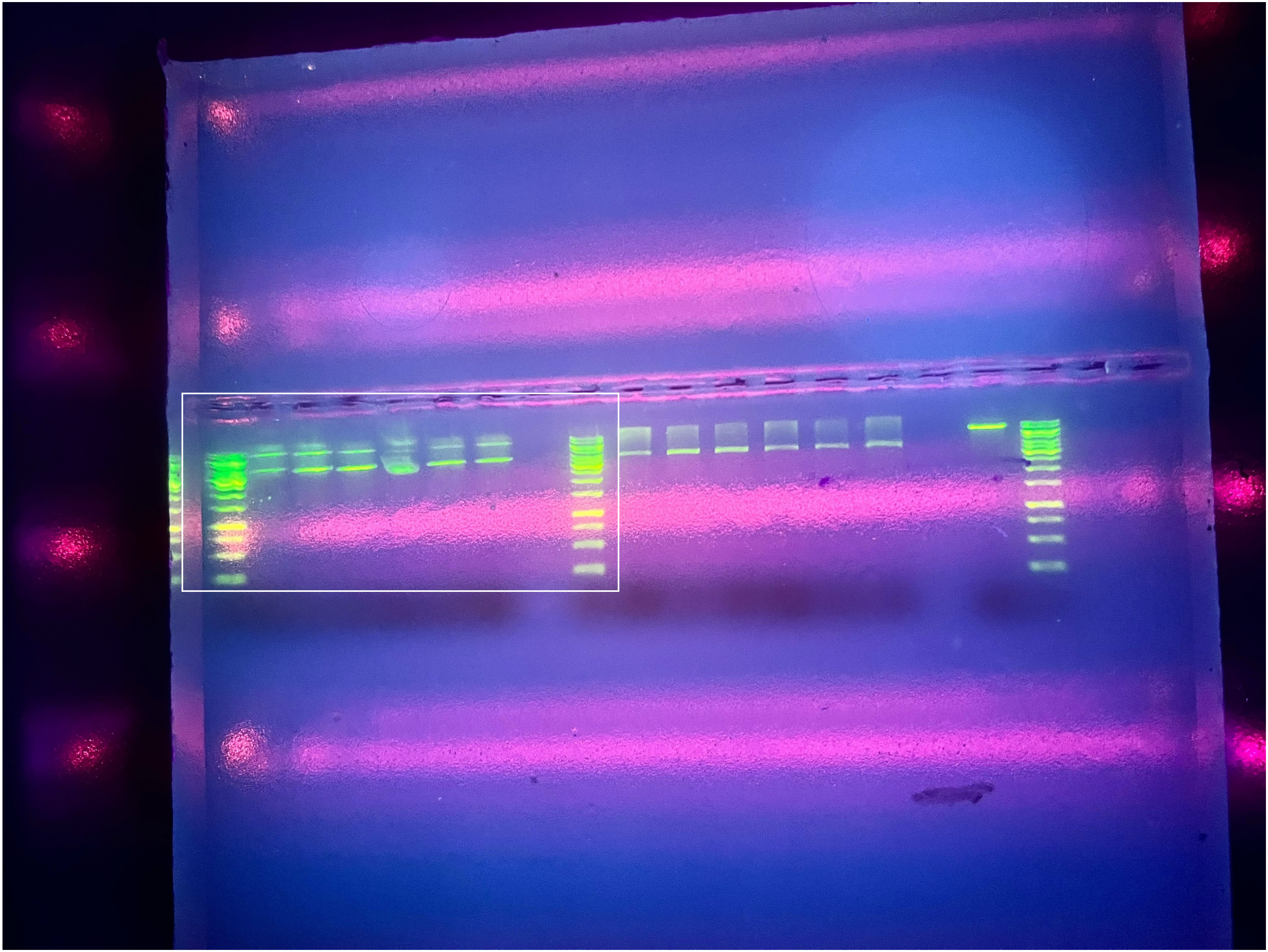

Supplemental Figure 1: Whole gel image for unacceptable minicircle elution
The white box is what was used for Figure 2, related to step 19.

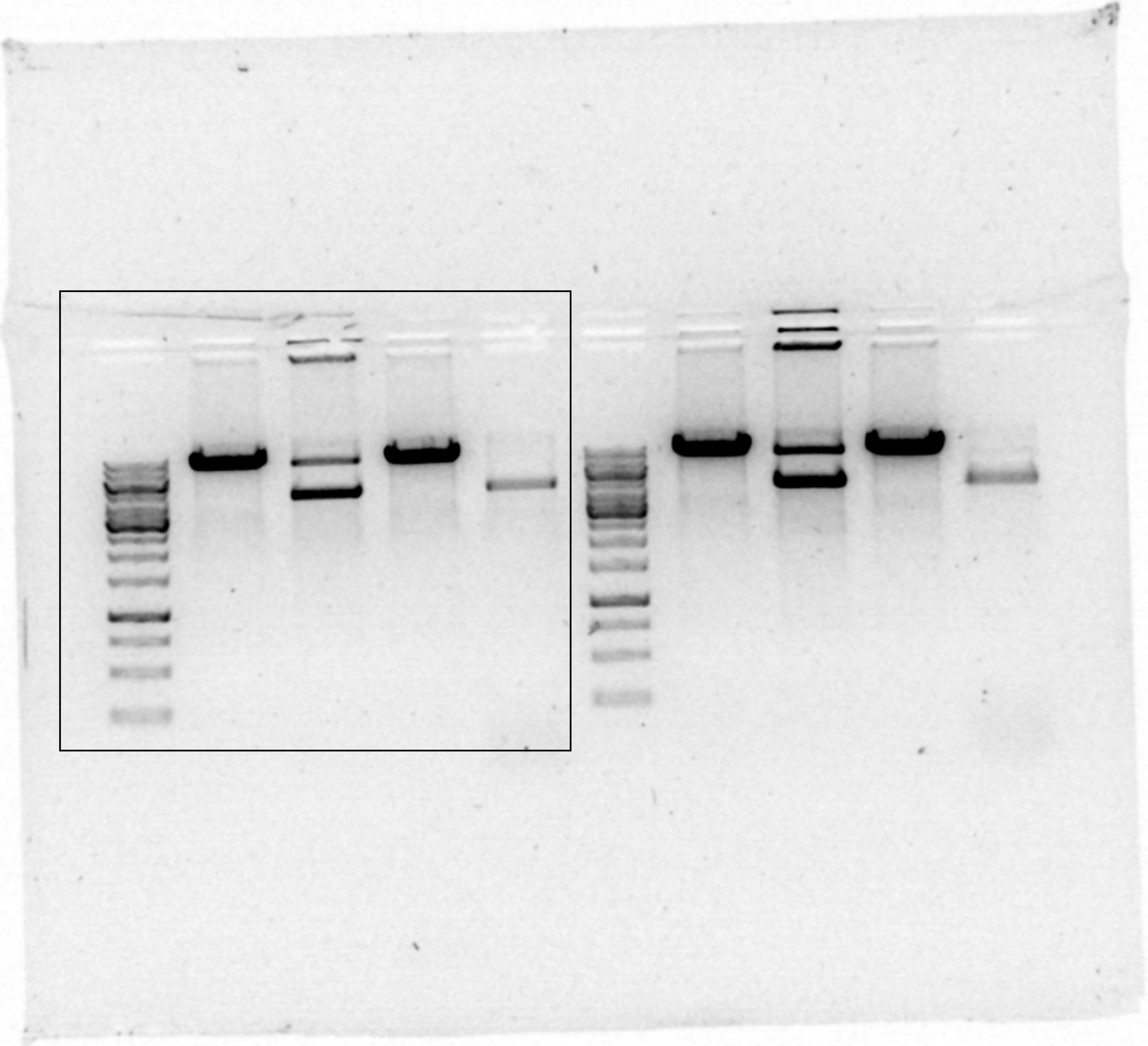

Supplemental Figure 2: Whole gel image for old vs new minicircle protocol comparison
The black box is what was used for Figure 3, related to expected outcomes.

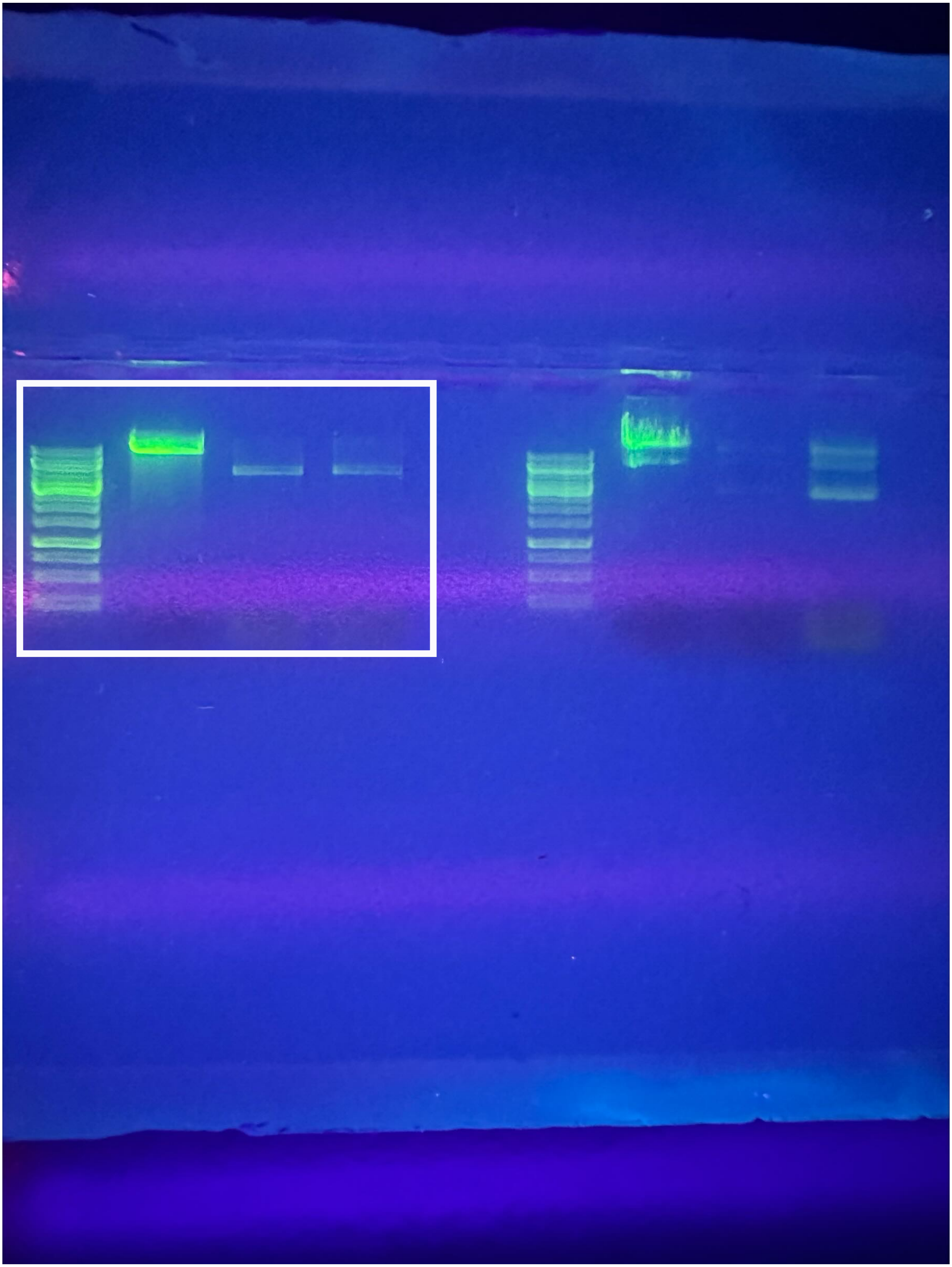

Supplemental Figure 3: Whole gel image for parental plasmid and minicircle elutions
The white box is what was used for Figure 4, related to expected outcomes.
